# Methodology to enable high-throughput imaging of Arabidopsis seedlings on cover glass-bottom multiwell plates

**DOI:** 10.17912/micropub.biology.001255

**Published:** 2024-09-25

**Authors:** Elena Mellado-Ortega, Hongwei Jing, Edward Wilkinson, Maria Lois, Lucia Strader

**Affiliations:** 1 Duke University, Durham, North Carolina, United States; 2 Center for Research in Agricultural Genomics, Barcelona, Catalonia, Spain; 3 Consejo Superior de Investigaciones Científicas, Madrid, Madrid, Spain

## Abstract

High-throughput imaging enables rapid collection of large datasets and is used widely in many systems. However, this is not often used in plant-based systems due to issues related to the need to mount tissues and autofluorescence of plant metabolites. We therefore developed methodology enabling high-throughput imaging of Arabidopsis roots. In this system, growth media supplemented with India Ink (to block autofluorescence from cotyledons) is poured directly into multi-well coverglass-bottom plates and seedlings grown such that the roots grow down with the gravity vector and along the coverglass, effectively mounting themselves for imaging. This method enables high-throughput imaging of Arabidopsis roots.

**Figure 1. A system for high-throughput imaging of Arabidopsis roots:**
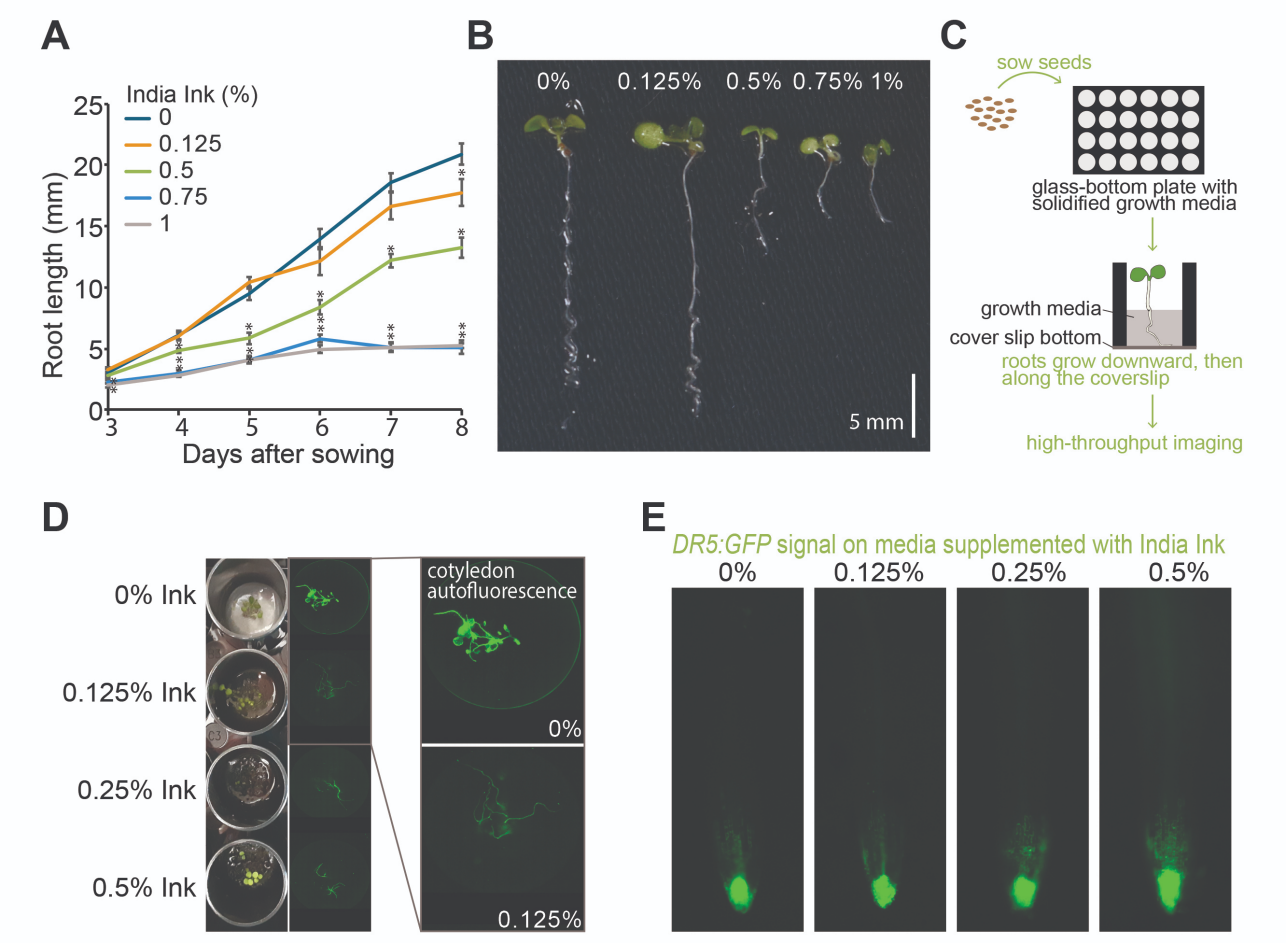
(A) Mean (± SE) root lengths of wild-type (Col-0) seedlings after 8 days of growth in magenta vessels with plant nutrient media supplemented with 0%, 0.125%, 0.5%, 0.75% or 1% India Ink (n=15). Asterisks indicate a statistically significant difference in root length when compared to mock treatment using a Two-tailed T-test assuming unequal variation (* = p ≤ 0.05). (B) Representative image of wild-type (Col-0) seedlings after 8 days of growth in magenta vessels with plant nutrient media supplemented with 0%, 0.125%, 0.5%, 0.75% or 1% India Ink. (C) Schematic of the experimental setup. Plant growth media is poured into coverglass-bottom multiwell plates. Once solidified, sterilized Arabidopsis seed is plated on the media surface and plates incubated in a plant growth incubator. The roots of the germinated seedlings grow downward with the gravity vector and follow the surface of the coverglass. Images are then acquired with an inverted fluorescence microscope. (D) Images of 8-day-old seedlings carrying *DR5:GFP* and grown under the indicated India Ink level in coverglass-bottom 24-well plates. Left, top view of plate. Right, bottom view of fluorescence images. Out-of-focus light from cotyledon autofluorescence is increasingly blocked with increased ink. (E) Arabidopsis roots expressing DR5:GFP on media supplemented with 0%, 0.125%, 0.25% or 0.5% India Ink.

## Description

Fluorescence microscopy is a well-established research tool in the life sciences (Lichtman and Conchello 2005) . Advances in microscopy techniques over the past 20 years have helped cell biologists address complex questions that cannot be answered using conventional biochemical assays. Although most imaging technology advances have been developed to image animal cells, several relevant applications in plant science have been developed (Busch et al., 2012; Komis et al., 2015; DeVree et al., 2021; Somssich, 2022) . These advancements have revolutionized diverse areas, such as large-scale organ morphogenesis, hormone signaling, and plant pathogenesis among others.

The need to image live plant cells in intact tissues raised the demand for developing novel experimental tools and setups (Shaw and Ehrhardt 2013) . Plants contain a myriad of autofluorescent molecules, among which the best known are pigments, such as chlorophyll and carotenoids, but also include secretory compounds and structural components of cell walls (reviewed in Donaldson, 2020); this inherent autofluorescence results in non-specific background fluorescence and presents a challenge for imaging. Another issue related to live cell imaging is light scattering that is caused by cell walls. This limits high-resolution approaches, a greater sensitivity and depth in the acquisition of images. Additionally, plants constantly respond to environmental stimuli. Therefore, tropic responses, such as to light and gravity, have to be considered to establish growth systems that allow non-invasive, high-quality imaging of intact and growing plant organs, such as roots (Grossmann et al., 2018) .

Widefield fluorescence microscopy is particularly useful for screening large samples and provides a flexible system for live cell imaging to monitor processes in real time. In these systems, image acquisition is fast and low cost, enabling the screening of large numbers of samples. However, widefield microscopy faces the drawback of having the entire specimen exposed to light, hindering image quality, particularly in plant specimens.

The peak of autofluorescence from chlorophyll is in the 714-725 nm and 735-745 nm range (Krause and Weis, 1991) . Despite this peak in the red range, chlorophyll autofluorescence emits sufficient light to interfere with imaging in other wavelengths, limiting many microscopy approaches.

To develop methodology that would enable high-throughput imaging of Arabidopsis roots, we wondered whether an opaque material, such as India Ink, could block the chlorophyll autofluorescence light. With the aim of preserving the biological integrity of the specimen at the same time that acquires images at a sufficiently high signal-to-noise ratio, we tested here a method in which plant growth media is supplemented with this material. This media is poured directly into multi-well coverglass-bottom plates, allowing the seedlings to grow such that the roots grow downward with the gravity vector and along the coverglass, effectively mounting themselves for imaging.

Firstly, to test whether India ink is a suitable material for carrying out microscopy-based multi-well assays, we evaluated the root development of Arabidopsis seedlings growing in magenta vessels for 8 days after sowing (Fig.1A). When the PNS media was supplemented with 0.125% of India Ink, we found no dramatic effect on root elongation during the first 5 days. After 8 days of growth, a 15% reduction in root length was evident compared to 0% ink. In contrast, the high levels of India Ink significantly inhibited root growth ( Fig. 1A and B). The two highest ink levels tested, 0.75% and 1%, produced similar and dramatic effects on root development. In the presence of these ink concentrations, on day 6 of growth, the root reached a length of 5 mm (one third of the control) and no further growth was observed on subsequent days. Therefore, these percentages were ruled out for the next imaging experiments.

According to these results, we further examined the effects on widefield light microscopy settings. For this, we examined the DR5:GFP reporter line, which monitors the transcriptional output of the phytohormone auxin (Ulmasov et al., 1997; Sabatini et al., 1999, review in Jedlicková et al., 2022). Seeds were placed in coverglass-bottom microtiter plates containing growth media supplemented with 0, 0.125%, 0.25%, or 0.5% India Ink ( Fig. 1D -E). Seedling images were taken at 8 days after sowing. In the absence of India Ink, chlorophyll autoflorescence transmits through the clear growth media and obscures the root signal of DR5:GFP ( Fig. 1D ). Inclusion of 0.125% India Ink effectively blocks autofluorescence (Fig 1D, right). Further, the 0.125% India Ink did not noticeably alter the DR5:GFP fluorescence signal (Fig 1E), suggesting that this amount of ink can be used in our experiments.

Live imaging is a powerful tool for understanding the dynamics of the biological processes that take place in living cells and organisms. Scientists often aim to recapitulate natural conditions in a laboratory setting. This data demonstrates a minimally invasive imaging method with sufficient spatial-temporal resolution to interrogate biological events as they occur. Our data indicate that adding a low percentage of India ink, such as 0.125%, to the plant growth media is an efficient method blocking autofluorescence from seedling cotyledons, improving the observation of root cells. Additionally, this opaque material, used at a low level, minimally interferes with Arabidopsis root growth in both multi-well plates and magenta vessels. The methodology described here is useful in high-throughput screenings based on widefield fluorescence microscopy assays aiming to identify bioactive compounds from large chemical libraries or to perform physiological studies.

## Methods

**Plant lines and growth conditions**

Root growth was examined from *Arabidopsis thaliana* accessions Colombia (Col-0). Seeds were surface sterilized with a solution of 20% (v/v) bleach and 0.01% (v/v) Triton X-100. After rising 4 times with sterilized water, the sterilized seeds were suspended in 0.1% agar and stratified for 2 days at 4 °C. Stratified seeds were plated in Magenta vessels (Merck KGaA, Darmstadt, Germany) containing plant nutrient media (PN) (Haughn and Somerville 1986) solidified with 0.6% agar and supplemented with 0.5% (w/v) sucrose. The indicated amounts of India Ink were added as a percentage of total volume. Seedlings were grown at 22 °C under continuous white light (GE fluorescent lamp (F17T8/SP41/ECO) at 120 µmol m ^-2^ s ^-1^ ) for 8 days. For imaging experiments, stratified seeds from *Arabidopsis thaliana* Col-0 carrying DR5:GFP (Ulmasov et al., 1997; Sabatini et al., 1999, review in Jedlicková et al., 2022) were plated in 24-well coverglass-bottom plates (Cellvis, Sunnyvale, California, USA) which contained PNS medium supplemented with the indicated concentrations of Speedball Super Black India Ink (Speedball).

For root elongation analysis, seedlings were photographed with a Nikon D810 digital camera (Nikon Corp. Japan) and root length was measured in ImageJ/FIJI (Schindelin et al. 2012) . Mean values were obtained from 15 replicates **.**

**Fluorescence imaging**

Imaging of the 8-day-old roots of DR5:GFP seedlings was performed with the DMI8 Leica widefield inverted microscope (Leica Microsystems, Germany), equipped with a DFC9000GTC-VSC12327 camera and DFT51010 quad filter block. GFP was excited with a 475 nm LED light source (excitation range 462-496 nm) and emission collected through the BP510/40 filter (emission range 506-532 nm). Images were acquired through a HC PL FLUOTAR 10X/0.32 NA DRY (Leica) objective.

## Reagents

Speedball Super Black India Ink (Speedball)
